# A modelling tool for policy analysis to support the design of efficient and effective policy responses for complex public health problems

**DOI:** 10.1186/s13012-015-0221-5

**Published:** 2015-03-03

**Authors:** Jo-An Atkinson, Andrew Page, Robert Wells, Andrew Milat, Andrew Wilson

**Affiliations:** Research Fellow, The Australian Prevention Partnership Centre, Sax Institute, PO Box K617, Haymarket, Sydney, NSW 1240 Australia; Professor of Public Health, School of Science and Health, University of Western Sydney, Campbelltown Campus, Penrith, NSW 2571 Australia; Deputy CEO, Sax Institute, PO Box K617, Haymarket, Sydney, NSW 1240 Australia; School of Public Health, University of Sydney, Edward Ford Building (A27), Camperdown, NSW 2059 Australia; Director, The Australian Prevention Partnership Centre, Sax Institute, PO Box K617, Haymarket, Sydney, NSW 1240 Australia

## Abstract

**Background:**

In the design of public health policy, a broader understanding of risk factors for disease across the life course, and an increasing awareness of the social determinants of health, has led to the development of more comprehensive, cross-sectoral strategies to tackle complex problems. However, comprehensive strategies may not represent the most efficient or effective approach to reducing disease burden at the population level. Rather, they may act to spread finite resources less intensively over a greater number of programs and initiatives, diluting the potential impact of the investment. While analytic tools are available that use research evidence to help identify and prioritise disease risk factors for public health action, they are inadequate to support more targeted and effective policy responses for complex public health problems.

**Discussion:**

This paper discusses the limitations of analytic tools that are commonly used to support evidence-informed policy decisions for complex problems. It proposes an alternative policy analysis tool which can integrate diverse evidence sources and provide a platform for virtual testing of policy alternatives in order to design solutions that are efficient, effective, and equitable. The case of suicide prevention in Australia is presented to demonstrate the limitations of current tools to adequately inform prevention policy and discusses the utility of the new policy analysis tool.

**Summary:**

In contrast to popular belief, a systems approach takes a step beyond comprehensive thinking and seeks to identify where best to target public health action and resources for optimal impact. It is concerned primarily with what can be reasonably left out of strategies for prevention and can be used to explore where disinvestment may occur without adversely affecting population health (or equity). Simulation modelling used for policy analysis offers promise in being able to better operationalise research evidence to support decision making for complex problems, improve targeting of public health policy, and offers a foundation for strengthening relationships between policy makers, stakeholders, and researchers.

## Background

The use of research evidence to underpin public health policy arose from a desire to improve the effectiveness of policy and population-level interventions. It was one response to criticisms of ineffective policies driven by crisis management, political objectives, and the lobbying of organised interest groups [1,2]. Government leaders in the United States, Canada, United Kingdom, and Australia have supported the increased use of evidence in public health policy [3]. However, its use to inform the development of effective policy responses to address complex public health problems presents both analytic and design challenges.

Over the past few decades, advances in life course epidemiology and an increasing awareness of the social determinants of health have revealed complex causal pathways to chronic illness [4], and broadened the range of factors that need to be considered in order to prevent non-communicable disease. Quantitative analytic tools commonly used to synthesise available evidence and assist with identifying and prioritising risk factors for public health action include systematic review with meta-analysis and calculations of population attributable risk. While these tools have no doubt provided valuable guidance to inform the development of effective public health policy, their adequacy and accuracy are called into question when applied to complex problems. This paper highlights the constraints of the traditional analytic approach (i.e. linear regression modelling) to accurately determine the strength of associations between risk factors and conditions that exhibit the characteristics of complexity, undermining confidence in the results of meta-analyses and calculations of population attributable risk that might have assisted in identifying how to best to target policy responses. Unsurprisingly, these challenges have contributed to the development of more comprehensive, cross-sectoral strategies to tackle complex public health problems in the hope that if risk factors are more comprehensively included in strategies for prevention, they are more likely to be effective. However, comprehensive strategies may not represent the most efficient or effective approach to reducing disease burden at the population level. Rather, they may act to spread finite resources less intensively over a greater number of programs and initiatives, diluting the potential impact of the investment. In addition to the analytic limitations of traditional tools to prioritise risk factors for optimal public health action, they can lack the ability to adequately inform policy responses. Policy responses often require a multidimensional design. This might involve legislation, regulation, enforcement, cross-sectoral cooperation, incentives, attempts at shifting sociocultural norms, and programs and services made up of multiple packaged interventions requiring supportive financial mechanisms, infrastructure, workforce, and governance structures to be integrated into complex, dynamic health and political environments [5]. Therefore, the synthesis of available evidence on the effectiveness and cost-effectiveness of interventions may not provide adequate guidance for the many questions that decision makers encounter as they attempt to design effective policy responses [5].

The case of suicide prevention in Australia is presented to demonstrate the lack of impact of the ‘comprehensive’ strategy on suicide rates over the last two decades, the limitations of traditional analytic tools, and to explore the potential benefits of systems science tools that can support the design of effective policy responses for suicide prevention and other complex public health problems.

## Discussion

### The complex problem of suicide

Suicide remains the leading cause of death in adult males aged under 44 years and women under 34 years [6] despite significant declines in young adult males since the 1990s [7], and represents significant economic and health service costs to Australia [8]. The rate of suicide in males (16.4 deaths per 100,000 population) was almost four times higher than in females (4.8 deaths per 100,000 population) in 2010 [6]. Many more females than males have attempted suicide despite their death rate being considerably lower [9]. The rate of suicide for Aboriginal and Torres Strait Islander peoples was twice that of non-Indigenous people over the period of 2001–2010 [6]. These figures do not include attempted suicide or other forms of deliberate self-harm.

Explanation and prediction of suicide remains immensely difficult due to its complex aetiology involving social, economic, cultural, interpersonal, and individual-level antecedents. Mental disorder is key risk factor for suicide [10,11] and has been identified as a National Health Priority Area in Australia [12]. Consequently, mental disorders often are the main focus of suicide prevention strategies [13]. However, other important determinants of suicide have been identified and range from proximal causes such as personal characteristics (i.e. biologic, genetic, cognitive, personality factors, sexual orientation, family history) [14-17], behavioural factors [18,19], and adverse life circumstances [20,21], to distal causes including macro-social and economic factors (i.e. segregation, unemployment, educational attainment, media) [11,22,23], cultural influences [24], and structural factors (e.g. economic policy, regulation of the means of suicide) [25-27]. There is a complex, dynamic interrelation of these factors across the life course and heterogeneity in their distribution among the population. This represents a significant challenge for researchers trying to establish causal relationships, and for policy makers at national and state level to determine how best to direct investment in suicide prevention initiatives.

Five major domains of suicide prevention interventions were identified by an international consortium of suicide experts as part of the most recent systematic review of the effectiveness of suicide prevention strategies [28]. These domains included the following: education and awareness programs for the general public and professionals; screening methods for high-risk persons; treatment of psychiatric disorders and follow-up care for suicide attempts (including pharmacotherapy and psychotherapy); restricting access to lethal means; and media reporting of suicide [28]. The review identified two suicide prevention approaches that were effective in reducing suicide rates: restriction of access to lethal methods, and physician education in depression recognition and treatment [28]. However, the sufficiency of this information for informing the design of policy responses for suicide prevention in Australia is limited. Questions remain as to whether a lack of evidence of effectiveness of other interventions is due to inadequate intensity and duration of implementation; the limited timespan for evaluation follow-up (as longer term trends in suicide rates are not captured in these studies); and how to interpret contextual variations in outcomes even within the same country [28]. In addition, such reviews can overlook the impact of targeting factors that may have a significant, but indirect effect on suicide rates such as occupational status and educational attainment [11,22].

### Policy response to the problem of suicide in Australia

In response to a significant rise in young male suicide between the 1970s and late 1990s, the Australian Government implemented the first National Youth Suicide Prevention Strategy in 1995 [29,30]. This strategy was expanded to all age groups with the launch of the National Suicide Prevention Strategy (NSPS) in 1999 and subsequent release of the LIFE Framework (Living Is For Everyone) in 2000 [31]. This framework took a comprehensive approach to addressing risk factors for suicide. Based on the LIFE Framework, over the next 6 years most States and Territories also adopted their own suicide prevention strategies [32]. During the 1999–2006 phase of the NSPS, over 150 community projects (mostly small-scale targeted programs with non-recurring funding) and 27 national initiatives were funded [32] which appeared not to have a major impact on the youth suicide rate [29].

In response to growing public and political concern that mental health reform was failing to achieve impact, two reports were released on the status of mental health services in Australia; *Not For Service: Experiences of injustice and despair in mental health care in Australian* (2005) [33] by the Mental Health Council of Australia; and the Senate Inquiry and report; *A national approach to mental health - from crisis to community* (2006) [34]. In 2006, the Council of Australian Governments (COAG) agreed to the National Action Plan on Mental Health 2006–2011 [35], which included a commitment by the Commonwealth Government to double funding for suicide prevention through the National Suicide Prevention Program from $62 million to $127 million [30]. In 2008, the Australian Suicide Prevention Advisory Council was established to provide national leadership and expert advice to the Australian Government as well as support the government’s implementation of the National Suicide Prevention Program. In response to further criticism that government efforts had resulted in fragmented services for those at risk of suicide, a further Senate Committee report (*The Hidden Toll: Suicide in Australia*) made recommendations to deliver more comprehensive and effective suicide prevention responses [30]. A sustained multilevel approach was recommended with efforts ranging from government to community responses, a combination of targeted and population-based interventions, underpinned by best available evidence [30]. Another key recommendation was increased coordination and alignment of suicide prevention programs and services in order to prevent overlap, duplication, and access gaps [30]. To implement these recommendations, the Australian Government released the *Mental Health: Taking Action to Tackle Suicide* package providing an additional $274 million over 4 years commencing 2010–2011 [36]. In addition to national and state government investments, business, community, and philanthropic funding sources also support suicide prevention programs and activities in Australia [30].

**Figure 1:**
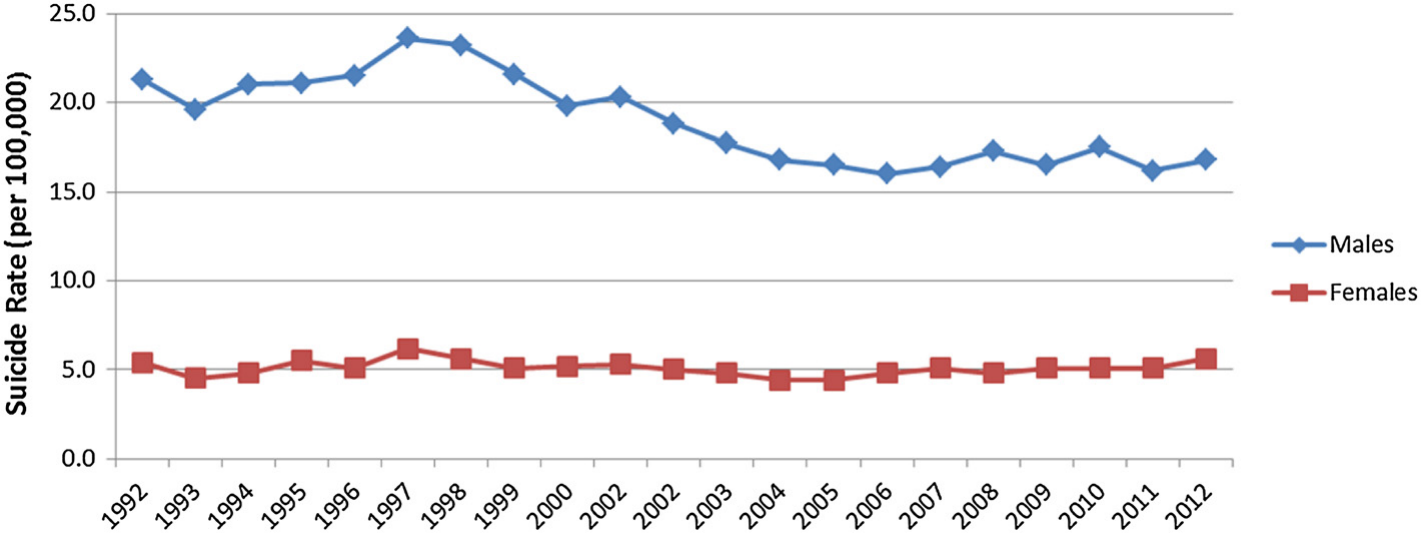
Suicide rates in Australia (1992–2012)*. *Data in this figure was obtained from Australian Bureau of Statistics (ABS) Catalogue 3303.0 Causes of Death Australia, 2012, released Friday 25th March 2014. For more information on data visit ABS website at www.abs.gov.au.

There is currently significant investment and policy momentum in suicide prevention in Australia. A large range of prevention programs and activities are being implemented at the national, state and local levels. These initiatives comprehensively address identified risk factors for suicide and include direct prevention initiatives (e.g. school-based interventions, targeted risk group initiatives, primary mental health care, early and crisis interventions, treatment and follow-up initiatives) and systems-level approaches (e.g. socioeconomic programs to mitigate risk factors, media education, restricting access to means of suicide, physical health promotion, and inter-sectoral collaboration) [37-40], but has this investment and activity to comprehensively address the range of risk factors for suicide had a significant impact on population-level suicide rates? Figure 1 demonstrates there was a decline in male suicide from 1997–2007. However, it is argued that these falls can be explained by a reduction in the availability of lethal methods of suicide, namely, measures to control the availability of firearms following the Port Arthur massacre, the requirement for new cars to be fitted with catalytic converters, and the decline in the prescription of tricyclic antidepressants (due to availability of a new class of antidepressant compounds with fewer side effects and lower toxicity in overdose) [41,42]. Therefore, the impact on male suicide was most likely a consequence of independent policy actions, unrelated to the national strategy for suicide prevention.

Over the same 10-year period, there was no decline in suicidal ideation or rates of attempted suicide [41]. This suggests that the root causes of suicide were not adequately addressed by the National Strategy, and while regulation of access to lethal means of suicide can reduce the death rate to a point, as an ongoing strategy, it may have limited impact due to the difficulty in regulating to prevent access to other suicide methods (hanging, sharp objects, jumping from a height).

This raises some important questions. Why is evidence of impact of the National Strategy for Suicide Prevention on population-level suicide rates limited? Do we really understand the complex and dynamic interrelation of causal factors of suicide over the life course? After almost 20 years of action, why are we still uncertain about how to effectively prevent suicide in Australia? If the ‘comprehensive approach’ to suicide prevention is not achieving impact, should we not consider alternative approaches? Unfortunately, current tools for synthesising and operationalising research evidence are not able to answer vital question of what the ideal targeting, intensity, consistency, and coordination of programs to prevent suicide is.

### Limitations of traditional analytic tools for supporting evidence-informed policy

For complex public health problems such as suicide, there are two important limitations of traditional analytic tools to support the design of effective evidence-informed policy responses:

- ***(I) Analytic limitations for exploring the impact of policy options***
- Evidence of measurable impacts of suicide prevention policy responses on population-level suicide rates is limited [28,29]. Uncertainties remain around the type, scope, and intensity of interventions to implement, and the right place and right period to implement them. Designing an effective and efficient policy response for suicide prevention requires a comprehensive perspective on causation, consideration of the influence of factors such as access to healthcare and preventive services, and analytic methods for testing the range of policy options and their consequences to better target actions for the Australian context. Numerous conceptual models of suicide have been developed for specific populations and stages of the life course [17,43-52], with varying emphasis on proximal causal factors, ecological influences, and multilevel determinants. While conceptual models can convey complexity, and map the interrelationships of multilevel factors, they cannot capture the magnitude of their influence (or temporal changes in influence over the life course) nor quantify the potential impacts of preventive interventions implemented individually or in combination, at various levels, using targeted and/or universal approaches.
- ***(II) Constraints of traditional approaches to data analysis***
- Dominant analytic methods attempt to identify the ‘determinants’ of an outcome and estimate the effect size of a given exposure/s on an outcome by controlling for common causes (confounders). Basic assumptions of this approach are that exposure variables (or ‘risk factors’) are independent, and relationships between exposures and outcome are unidirectional, linear, and constant through time. This approach does not necessarily capture health behaviours as being a result of interacting and interdependent ‘risk factors’ acting at multiple levels (e.g. individual characteristics, social networks, economic, and political environments). Nor does it reflect how these multilevel ‘risk factors’ shape one another, and in turn are shaped by health and health behaviours (i.e. relationships between variables can be characterised by interdependence, nonlinearity and feedback loops) [53]. These characteristics violate the conditions for use of traditional analytic methods. While traditional methods provide valuable data-driven explanations of simple causal relationships between a finite range of variables for well-defined problems, and rigorously take account of variables that can confound these relationships [53], public health problems that arise from complex human behaviours makes reliance on traditional methods problematic and undermines confidence in the ability of research evidence to inform effective policy.

### An alternative analytic tool offering promise for more efficient and effective evidence-informed policy

Systems science is an interdisciplinary field that investigates the nature of complex systems and is underpinned by well-established mathematical theory of nonlinear dynamics [54-56]. It is not a new science, and its methods have been successfully applied to sectors such as engineering, defence, economics, ecology, and business since the mid-1950s. Systems science is increasingly being recognised in the health sector for its utility in mapping and understanding complex health problems, operationalising research evidence, and systematically analysing a range of intervention and policy solutions [57]. System dynamics and agent-based modelling (simulation modelling) are systems science methods that can be used to develop a tool for policy analysis. Such a tool would allow virtual (desktop) experimentation of policy scenarios to test their comparative impact and cost over the short, medium, and longer term. The policy analysis tool could test the efficiency, effectiveness, and equity of policy responses, exposing unintended consequences and perverse incentives in the system through computer simulation, averting the need for costly trial and error approaches.

Systems science adopts a perspective that encompasses the inherent complexity of a public health problem and avoids inferences being drawn from narrowly focussed investigations. System dynamics and agent-based modelling take into account the interrelations, reciprocity, discontinuity, and dynamic nature of influences on health and health behaviours within a broader context [53]. The structure of relationships between numerous interacting factors is mapped and modelled encompassing feedback and delays [58]. This enables analysis and identification of causal loops that are most influential in determining the evolutionary behaviour of the system that produces the public health problem in question [58]. These systems science methods are therefore better able to embrace and make sense of the complexity that characterises public health problems such as suicide.

In particular, multiscale modelling has been successfully applied in biology, environmental sciences, and physical sciences, but only recently adapted to address public health problems [59]. It provides a method for mapping and understanding how proximal causal factors interact with each other and with ecological factors to determine health behaviour [53]. It combines agent-based modelling (capable of capturing heterogeneous attributes, behaviours, and interactions of individuals) and system dynamics modelling (which captures population-level, ecological influences, and whole system dynamics). Multiscale modelling provides policy makers with a powerful analysis tool that is also capable of exploring equity effects of policy scenarios.

In contrast to the common ‘comprehensive approach’ to addressing complex public health problems such as suicide, a systems science approach (while encompassing holistic, multiscale, cross-sectoral thinking) seeks to identify where best to focus public health action, and with what intensity, and is concerned primarily with what can be reasonably left out of strategies for prevention. That is, simulation modelling can be used as a policy analysis tool to inform more efficient targeting of resources at specific risk factors, using particular interventions and approaches projected to have greatest impact, while exploring where disinvestment can occur without adversely affecting population health (or equity). The potential utility of these systems science methods lie in their ability to systematically and quantitatively analyse a range of intervention and policy options and identify leverage points in the system (places to intervene) where small inputs might result in large impacts [60]. This alternative approach should be considered to support decision making for future strategies to address complex problems such as suicide prevention, particularly when substantial investments in a ‘comprehensive approach’ over many years has failed to yield significant impacts.

Simulation modelling commences with the collation of existing conceptual models of suicide, reviews of research evidence, and expert knowledge. Dialogues informed by the collated information proceed to conceptual mapping, quantification, and simulation. Models are able to incorporate the impact of contextual influences on policy making (e.g. political and economic environments, community sentiment). If desired, the process can permit the broader involvement of key stakeholders in model development which may act to foster trust and transparency in the policy-making process and accelerate policy adoption, implementation, and health sector change. Models are considered to be a theoretical representation of the complex problem and hence undergo a validation process that includes measuring how accurately the model can reproduce ‘real world’ historical data patterns. This important validation step helps build confidence in the structure and predictions of a model. The final product is a policy analysis tool that can systematically explore the impact on suicide of

- Individual interventions;
- Combinations of interventions applied simultaneously or sequentially;
- Interventions applied universally or targeted at particular groups;
- Changes to arrangements of the system relating to workforce, infrastructure, governance and financing;
- The different scenarios can be modelled over time, costed, and trade-offs explored.

Systems science methods are not without criticism. Instances where their use have relied heavily on experiential knowledge of stakeholders to map causal relationships between variables without recourse to evidence from the literature, has resulted in the perception of systems science as ‘soft science’, and the misconception that it rejects traditional scientific views [57]. In reality, empirical analyses of causation of system components, and simulation modelling that brings these components together positing their interaction, are complementary. Integrating the use of sound epidemiological approaches to specify the most likely causal pathways between putative exposures and outcomes, with simulation modelling to explore whole system dynamics, may contribute to the credibility of systems science methods. Of particular relevance in this regard is the use of directed acyclic graphs [61], which explicitly define the hypothesised causal relationships between exposures, confounders, intermediaries, and effect measure modifiers. These then guide the analytic strategy to obtain less biased estimates of an association between an exposure and outcome. These approaches have most commonly been used in the context of defining aetiological assumptions and analytic adjustments [61]. However, directed acyclic graphs and causal relationships which incorporate levels of scale inherent in eco-social approaches to understanding health could be adapted to inform the content of multiscale models and lead to more empirically defensible simulations of hypothetical impacts of interventions or public health policies.

### The potential of systems tools to support evidence synthesis and knowledge translation

The extent to which evidence is incorporated into public health policy is challenged by factors including the political environment [62,63]; vast, inconsistent, or inaccessible scientific information [62,64,65]; deficits in relevant and timely research [63,65]; a tradition of relying on intuition or advice from opinion leaders [62,66,67]; and inadequate information systems, resources, leadership, and required competencies to capture and synthesise disparate evidence sources [1,62,68-70]. The use of simulation modelling offers potential in addressing many of these challenges, particularly when policy makers and other key stakeholders are engaged in the model development process. For example, applications of this method have led to increased awareness by policy makers of the dynamics of the health problem to be addressed and demonstrated to them how research findings and data can be practically used to generate projections that can guide policy decisions (Atkinson J, Wells R, Page A, Dominello A, Haines M, Wilson A: A review of applications of system dynamics modelling to support health policy making, submitted). In addition, it provided a framework to facilitate more rapid integration and use of new evidence that arises for policy analysis, and it led to insights on the value of collaboration with non-health sectors. These benefits are an illustration of Weiss’ notion of ‘knowledge creep’ [71], where research enters the mental models of policy makers through the clash and compromise of ideas from diverse perspectives and the insinuation of research evidence into this melee [72]. Simulation modelling provides a platform for this process and for strengthening relationships among policy makers, stakeholders, and researchers.

In addition, a systematic narrative review of the influences on evidence uptake by policy makers found a recurring theme that the decision process is influenced by opinion leaders, who either make judgements based on expert opinion, or act as a filter through which research evidence is transferred, undermining its neutrality [66]. Simulation modelling can diffuse this process by providing a platform for systematic integration of diverse evidence sources and encourages participation of stakeholders in development of the model that is then used by policy makers as an impartial tool for analysis of policy options. This contributes to transparency in the decision-making process, fosters trust and greater buy-in by stakeholders, and can accelerate policy adoption, implementation, and change.

Table 1 summarises further benefits of using simulation modelling to operationalise research evidence for the development of policy responses for complex public health problems. However, further work is needed to adapt, apply, and evaluate the method as a knowledge translation tool to support evidence-informed public health policy and practice.

**Table 1:** **The benefits of using simulation modelling for the design and analysis of public health policy** [71,72]

**Benefits specific to knowledge translation**	**General benefits**
● Provides a framework for operationalising vast amounts of often inaccessible scientific information	● Assists with more systematic decision-making where there are evidence gaps
● Actively engages multi-disciplinary stakeholders in model design	● Elucidates leverage points in the system, where small inputs result in large impacts
● Facilitates the development of a common ‘mental map’ for progress and consensus on optimal policy decisions	● Guides prioritisation and planning for resource efficiency and simulates scenarios that can add strength to business case development
● Provides a formal channel for ongoing engagement and communication/information translation between researchers and policy makers as the model is updated to incorporate additional or changing evidence over time	● Provides a framework for future research and evaluation of policy implementation
● The model is available for routine use to simulate and analyse policy options/changes in a policy friendly timeframe	● Can capture complex influences on a particular public health problem including political factors (national mood; actions and reactions of powerful vested interests, e.g. lobbyists, advocacy groups to simulated policy decisions)
● Assists with countering the tradition of relying on intuition for policy decisions	● Can facilitate the identification of policy responses that have improved contextual orientation and increased effectiveness
● Can facilitate cross-sectoral communication and synthesis of knowledge	

#### Potential pitfalls

The recent systematic review of the application of system dynamics modelling for health policy making yielded only seven case studies, two of which described their use to address public health problems (Atkinson J, Wells R, Page A, Dominello A, Haines M, Wilson A: A review of applications of system dynamics modelling to support health policy making, submitted). The lack of published literature detailing practical applications of this methodology to support health policy making precludes a detailed understanding of the issues and limitations of the approach both as a knowledge translation tool and in the design of effective health policy. However, a systematic review and synthesis of 107 applications of system dynamics modelling in non-health sectors reported that among its many benefits, several potential pitfalls exist [73]. These include, a mismatch between the level of abstraction of the model and stakeholders’ mental models (i.e. the model was either too abstract or too big to understand), and the modelling techniques used did not match the project circumstances (i.e. use of unstructured discussion for mapping of a problem that is too politically sensitive or too broad to achieve focus) [73]. Such pitfalls can prevent the process of gaining insights into a problem, understanding the mental models of others, and ultimately achieving consensus on the structure and behaviour of the model. Without this consensus, the confidence of some key stakeholders in model projections and subsequent policy decisions will be lacking.

### Summary

Simulation modelling used as a policy analysis tool and based on a detailed understanding of aetiology and evidence-based estimates of the impact of preventive interventions on absolute and relative risk provides a rigorous mechanism for virtual testing of policy alternatives in order to determine which policy responses would achieve the greatest impact. It is an approach that offers promise in being able to better operationalize research evidence to support decision making for complex problems, improves targeting of public health action, and provides a platform for strengthening relationships between policy makers, stakeholders, and researchers.

Next steps for realising the benefits of this policy analysis tool to support evidence-informed policy responses in public health include leveraging existing structures and expertise; developing the necessary capacity and processes to support implementation of this approach in Australia; and evaluating their use. The recently established Australian Prevention Partnership Centre [74] is aiming to achieve this in the prevention of lifestyle-related chronic conditions through the use of systems tools and approaches. Among other outcomes, the Partnership Centre is working towards developing stronger national networks of researchers, policy, and program practitioners. This provides a valuable opportunity for co-production of simulation models to better inform policy responses for complex public health problems.
